# Process Development for the Instant Quantification of Lycopene from Agricultural Produces Using Supercritical Fluid Chromatography-Diode Array Detector (SFC-DAD)

**DOI:** 10.3390/foods11040522

**Published:** 2022-02-11

**Authors:** Supriya Priyadarsani, Avinash Singh Patel, Yogesh Sharma, Abhijit Kar

**Affiliations:** 1 Division of Food Science and Postharvest Technology, ICAR-Indian Agricultural Research Institute, New Delhi 110012, India; Spriyadarsani23@gmail.com (S.P.); avinash.patel@maine.edu (A.S.P.); 2 Engineering Division, ICAR-National Rice Research Institute, Cuttack 753006, India; 3 Department of Food and Nutrition Sciences, School of Food and Agriculture, University of Maine, Orono, ME 04469, USA; 4 Waters India Pvt. Ltd., New Delhi 110025, India; yogesh_sharma@waters.com

**Keywords:** lycopene, supercritical fluid chromatography (SFE), process optimization, validation and quantification

## Abstract

A quick, simple, and reliable isocratic ultra-performance supercritical fluid chromatography-photodiode array detector (UPSFC-DAD) method was developed and validated to determine lycopene in different horticultural products. The effects of stationary phase, co-solvent, pressure, temperature, flow rate, and mobile phase additive on the separation of lycopene were evaluated. The developed method involved BEH-2EP—2.1 × 150 mm, 5 µm as the stationary phase, and CO2/MeOH 85:15 (*v*/*v*) with formic acid as the additive at 0.10% as the mobile phase. The column temperature was maintained at 45 °C, ABPR at 1800 psi, and the mobile phase’s flow rate was maintained at 1 mL/min. Under the optimized conditions, lycopene was successfully separated within 0.722 ± 0.001 min. The standard curve assayed over a range of 10 to 100 µg/mL resulted in a correlation coefficient of 0.998. The mean recoveries between 97.38% and 102.67% at different spiking levels with RSD < 2.5% were achieved. The intra and inter-day precision expressed as relative standard deviations (RSD) were found to range from 1.27% to 3.28% and from 1.57% to 4.18%, respectively. Robustness in terms of retention time (tR) and RSD were found to be 0.93 ± 0.23 min and less <2.80%, respectively. The limits of detection and quantification were 0.14 µg/mL and 0.37 µg/mL, respectively. This method was successfully applied to determine lycopene extracted from papaya, grapefruit, and bitter melon.

## 1. Introduction

Lycopene is an acyclic, open chain, unsaturated carotenoid with 13 double bonds, 11 of which are conjugated and arranged in a linear array. It is one of the most promising bioactive compounds because of its potential to inhibit invasion of free radicals at the membrane surface, which triggers the primary defense mechanism of the human body against several chronic diseases [1,2,3,4,5]. It is also considered to be a potent anticarcinogen and an antioxidant [6,7]. Lycopene plays a significant role in mitigating oxidative stress as it lessens the inducible nitric oxide synthase activation [8]. Studies depict lycopene as a phytochemical that can play a significant role in cancer prevention and cardiovascular health [6,8,9,10]. Several recent studies have been conducted among the carotenoids utilizing lycopene for food applications, such as food emulsion, extraction for fortification, and lipid oxidation [11,12,13,14,15].

Metabolic profiling of carotenoids is necessary for understanding the functionality of the compounds, which is why advanced technologies are being used to keenly analyze these samples. 

RP-HPLC coupled with UV–Vis absorbance detection or diode array detection (DAD) and C18 column are most commonly used to measure lycopene in dietary supplements, with a separation time of 14.2 min [16,17]. A study was conducted by Bijttebier et al. (2014) to separate complex carotenoids mixtures using HPLC C30 column and compare it with other columns. It was found that HPLC 30 performed better in terms of carotenoids separation as compared with other columns. Nevertheless, the only drawback was the long analysis time, which indicates more consumption of solvent, which is non-environment-friendly and not found to be suitable for unstable compounds [18].

The growing importance of lycopene, coupled with its extreme non-polarity and susceptibility to heat damage, necessitates the development of a specific, reliable, and quick method of determination [19]. Ultra-performance supercritical fluid chromatography (UPSFC) is a separation science category that uses a supercritical fluid as a mobile phase and provides orthogonal and increased separation power, compared with liquid or gas chromatography [20,21,22]. Low viscosity, high diffusivity, liquid-like solvation power, miscibility with entire series of eluotropic solvents, inert, non- flammable, and non-toxic nature of the supercritical carbon dioxide makes it the most prevalent mobile phase in supercritical fluid chromatography [23,24]. The mobile phase diffuses into the sample matrix during supercritical chromatography, minimizing the column’s pressure drop by many folds [25]. It leads to easy separation and isolation of chiral and achiral molecules [26,27]. The supercritical mobile phase’s elution strength is often enhanced by adding polar organic solvents such as methanol, ethanol, and others as a modifier for analyzing polar compounds [28,29,30].

Li et al. (2015) investigated the separation of nine selected carotenoids, including lycopene, α- carotene, β-carotene, β-cryptoxanthin, astaxanthin, fucoxanthin, canthaxanthin, lutein, and zeaxanthin in dietary supplements, such as tablet or capsule using ultra-high performance supercritical fluid chromatography attached to a photodiode array detector (PDA) and sub 2 µm particle columns (HSS C18 SB column). The combination of 1:2 (v:v) methanol/ethanol mixture, column temperature of 35 °C, and a backpressure of 15.2 MPa successfully separated the carotenoids in 10 min with R^2^ = 0.997 [31].

Supercritical fluid chromatography (SFC) coupled with mass spectrometry (MS) involved an octadecyl-bonded silica (ODS) particle-packed column that was used for separation of seven carotenoids, including structural isomers in green algae, which resulted in complete separation within a time interval of 15 min using the combination of pressure of nearly 2600 psi, flow rate of 3 mL/min, and column temperature of 35 °C. [32]

In a study conducted by Guedes et al. (2017), UPSFC was successful in separating lycopene, β-carotene, coenzyme Q10, and lutein at the optimum operating parameters of pressure of 1500 psi, ethanol percentage of 15%, and temperature of 25 °C in palm oil. [33]

In this study, we aimed at developing and validating an individual, fast, reliable, selective, economic, and, most importantly, a sustainable method for quantifying lycopene into underutilized agricultural products using UPSFC coupled with a photodiode array (PDA) detector and suitable solid phase. It includes screening different stationary and mobile phases following flow rates, pressure, and the UPSFC system temperature.

## 2. Materials and Methods

### 2.1. Chemicals and Reagents

**Figure 1:**
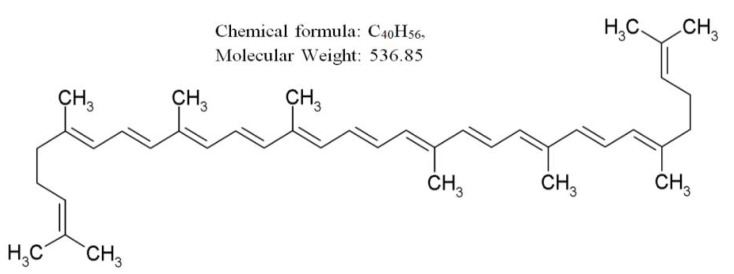
Chemical structures of lycopene.

Reference standard lycopene (≥90%) was purchased from Sigma-Aldrich (Munich, Germany). The chemical structure of lycopene is given in Figure 1. HPLC grade acetone, petroleum ether, ethanol, methanol, tert-butyl methyl ether (TBME), and n-hexane were purchased from Merck (Darmstadt, Germany). Analytical grade anhydrous sodium sulphate was purchased from Sisco Research Laboratories Pvt Ltd. Compressed liquid CO_2_ (99.99%) cylinders were purchased from Amit Labs (New Delhi, India).

### 2.2. Instruments and Chromatographic Conditions

UPSFC-DAD analysis was performed using a Waters Acquity UPC2 system consisting of a binary solvent delivery pump, an auto sampler, a column oven, an automated back pressure regulator, and a photodiode array detector (all from Waters Corp., Milford, MA, USA). Empower software (version 3) Build 3471 @copyright 2010 Waters Corpration, USA was used for instrument control and data acquisition. Methanol/TBME (1:1 *v*/*v*) was used for strong needle wash, soft needle wash, and seal wash.

### 2.3. Preparation of Lycopene Stock and Working Solution

Lycopene stock solution (200 ppm) was prepared by diluting 1 mg of lycopene in 5 mL n-hexane and transferring it to an amber-colored volumetric flask to prevent light degradation. Moreover, working solutions of 10 to 100 µg/mL were prepared by diluting the stock solution using absolute n-hexane.

### 2.4. Scouting or Method Optimization

Scouting is defined as an essential step for method development. It is mostly carried out to screen different modifiers on several columns packed with the stationary phase. Optimization of the chromatography parameters for using UPSFC-DAD was performed by the method as designed by Ashraf-Khorassani et al. [34]. Suitability of the available stationary columns named: (1) BEH—3 × 100 mm, 1.7 µm; (2) C18—3 × 100 mm, 1.8 µm; and (3) BEH-2EP—2.1 × 150 mm, 5 µm—with an isocratic elution of a combination of two mobile phases CO_2_/methanol (MeOH) and CO_2_/ethanol (EtOH) at different ratio of 100:0; 90:10; 85:15, and 80:20 (*v*/*v*) were maintained. During the screening of stationary and mobile phases, flow rate, automated back pressure regulator (ABPR), and temperature of the system were fixed at 1.0 mL/min, 1800 psi, and 45 °C. The spectra of the lycopene were collected at 434 nm over 2 min of the total running period. Once the stationary and the combination of mobile phases were screened, the effects of other process parameters—such as flow rate of 0.5, 1.0, and 1.5 mL/min; ABPR of 1600, 1800, and 2000 psi; and column temperature of 35 °C, 45 °C, and 55 °C—were also optimized to improve the characteristics of the lycopene chromatograph.

### 2.5. Computation of Chromatographic Parameters

Retention time (tR), tailing factor (T), retention factor (k), theoretical plates (N), height equivalent to a theoretical plate (HEPT), and reduced plate height (h) are a few parameters that can be effectively used to understand, compare, simplify, and describe the principles of chromatography [35]. The time taken for a particular compound to travel through the column to the detector is known as its retention time (tR). It is measured from when the sample is injected to the point at which the display shows a maximum peak height for that compound. The tailing factor is a measure of peak tailing. It is the distance from the front slope of the peak to the back slope divided by twice the distance from the centerline of the peak to the front slope, with all measurements made at 5% of the maximum peak height [36].

The retention factor (k) is a measure of a sample molecule’s retention time, relative to dead column volume (V_0_).

(1) $$k=\frac{V_{1}-V_{0}}{V_{0}}=\frac{t_{1}-t_{0}}{t_{0}}$$

where k is the retention factor of the column; V_0_ is the void volume (dead volume) of the column (volume at which an unretained component elutes); V_1_ is the retention volume of peak 1. If the flow rate is constant, they can also be reported by their respective retention times; t_0_ is the time at which unretained component elutes, and t_1_ is the retention time of peak 1.

The chromatographic number of theoretical plates (N) was calculated using the equation given below:

(2) $$N=16{\left(\frac{V_{e}}{W_{b}}\right)}^{2}$$

where Ve is the elution volume, and W_b_ is the width of the peak at baseline.

Van-Deemter [37] described the height equivalent to a theoretical plate (HEPT), which was calculated to compare between different columns using the given equation:

(3) $$\mathrm{HEPT}=\frac{L}{N}$$

where L is the length of the column, and N is the number of theoretical plates.

And finally, the reduced plate height (h), which is a dimensionless parameter that allows the direct comparison of the efficiency of two or more columns packed with different particle size packing materials, was calculated using the given equation:

(4) $$h=\frac{\mathrm{HEPT}}{d_{p}}$$

where d_p_ is the mean particle size (µm) of the column.

### 2.6. Optimization

The fresh papaya, grapefruit, and ripe bitter melon were collected from the local orchard of the Indian Agriculture Research Institute, New Delhi, India. The aril from the ripe bitter melon was manually separated by removing the outer pericarp and subsequently removing the seeds. The lycopene extraction was performed as the method described by Phinney et al. [38]. An amount of 5 g of papaya, grapefruit, and ripe bitter melon aril was taken for the extraction separately using mortar and pestle with acetone. An amount of 2 g of sea sand was used to facilitate the extraction. Extracted lycopene was purified using a separating funnel by adding 10 mL of petroleum ether, followed by 20 mL of 5% Na_2_SO_4_ solution. After a repeated extraction and purification process, the extracts were evaporated under a rotary evaporator (Heidolph, Germany) at room temperature in the absence of light. The vaporized sample was stored at −20 °C for further quantification using the developed UPSFC method. Samples were resolved in 3 mL n-hexane, filtered through a syringe driven filter unit (0.22 µm size, PVDF, Merck Millipore Ltd., Darmstadt, Germany). The filtered samples were transferred into pre-slit screw-capped, amber-colored vials and were placed on the autosampler 96-well plate (Waters Acquity UPC^2^).

## 3. Results and Discussion

### 3.1. Method Development and Optimization

#### 3.1.1. Influence of Stationary and Mobile Phase

Screening for stationary and mobile phases for the separation of lycopene was carried out on three types of available columns: (i) BEH—1.7µm; (ii) C18—1.8 µm, and (iii) BEH-2EP– 5 µm. Additionally, an isocratic elution of a combination of two mobile phases CO_2_/MeOH and CO_2_/EtOH at different ratios of 100:0, 90:10, 85:15, and 80:20 (*v*/*v*) were examined. Retention times (tR) and other chromatographic parameters influenced by stationary phases were recorded and calculated; results are given in Table 1. A combination of BEH 2-EP column (2.1 × 150 mm, 5 µm) as stationary phase and CO_2_:MeOH:: 85:15 was found to be the most suitable one because of its minimum T, HEPT, and h values and maximum k and N values. The retention time for this combination was found to be 0.72 min.

The tR was found to decrease with the column particle size decrease from 5 µm (BEH 2EP) to 1.7 µm (BEH and HSS C_18_). The decrease over BEH 2EP was 36.15% and 13.78%, respectively, for BEH and HSS C_18_ columns. Similar trends have been reported by Liu et al. [39], who found that the decrease in the particle size of the stationary phase generated more backpressure when the length and flowrate are constant. It was presumed that the stationary phase’s smaller particle size was because of its less porous structure than the column with larger particle size [40].

The organic modifier was frequently used in supercritical fluid chromatography (SFC) to modify the polarity mobile phase and improve composites’ solubility, which may otherwise tend to condense during the investigation period. An increased solubility permitted the improvement in separation by changing the solute–mobile phase interactions [41,42]. For all combinations of stationary and mobile phases, the addition of co-solvent reduced tR significantly. An increase in the co-solvent percentages further reduced the tR. From among the two co-solvents studied, methanol could reduce tR more (33.5% to 51.3%) than ethanol (19.8% to 38.2%). From among the solid phases used, HSS C_18_ had the maximum effect (38.22% to 48.48%), followed by BEH (19.79% to 51.29%) and BEH 2EP (23.52% to 33.53%). When a 1:1 (*v*/*v*) mixture of methanol and ethanol was used, all carotenoids showed complete baseline separation, the resolutions of astaxanthin and canthaxanthin and of canthaxanthin and fucoxanthin increased to 2.46 and 2.34, respectively in the dietary supplements (tablet or capsule) [31].

The tailing factor (T) was reduced with the column particle size (Table 1). The addition of co-solvent (ethanol and methanol) further reduced the tailing factors compared with those obtained using CO_2_ exclusively as the mobile phase (6.28% and 66.90% for BEH 1.7; 14.70% and 25.90% for HSS C_18_; and 35.49% and 57.84% for BEH 2EP). However, an increase in co-solvent beyond 15% increased the tailing factors for both of the co-solvents used in all three stationary phases. From among the two columns (BEH 1.7 and HSS C_18_) having the same particle sizes, HSS C_18_ was found to have lower tailing factors at all the eight mobile phase combinations.

The retention factor (k) decreased with increasing modifier concentrations beyond 15% (*v*/*v*), and the peaks were almost merged at a concentration of 20% (*v*/*v*). In contrast, the problem of broader peaks was observed when modifier concentration was decreased. Amongst all the combinations of stationary and mobile phases considered for an accurate and reliable elution of lycopene, BEH 2EP as a stationary phase combined with a binary mobile phase CO_2_/MeOH:: 85:15 (*v*/*v*) was found to have the k value of 0.632, respectively.

An increase in the number of theoretical plates (N) was observed with an increase in the column particle size (1837 for BEH 1.7; 2045 for HSS C_18_; and 4198 for BEH 2EP) when only CO_2_ was used as the mobile phase (Table 1). The addition of co-solvent (ethanol and methanol) further enhanced the number of theoretical plates. The values doubled with a 10% addition of methanol as co-solvent. They tripled when the percentage of the co-solvent increased to 15%, using methanol as a co-solvent and BEH 2EP as the stationary phase. Nonetheless, a further increase in the co-solvent to 20% (*v*/*v*) resulted in a decrease in the number of theoretical plates. The results were ascertained by comparing the heights equivalent to a theoretical plate (HEPT) between the different columns. With BEH 2EP and methanol as co-solvent/modifier, all percentages were found to have the lowest HEPT. A significant increase in the number of theoretical plates in the BEH 2EP column over those of BEH 1.7 and HSS C_18_ is likely to have been due to a better elution of lycopene resulting from its 30% reduced diameter and 50% increased length.

The reduced plate height (h) dimensionless parameter introduced by Lesellier [43] allowed a direct comparison of the efficiency of two or more columns packed with different particle size-packing materials. According to the theory proposed by Giddings, a well-packed column should have a reduced plate height (h), not exceeding 2 to 3 µm. From among the combinations of stationary and mobile phases studied, a minimum value of 3.97 was obtained for a variety of BEH 2EP as a stationary phase combined with CO_2_/MeOH: 85:15 (*v*/*v*) as the mobile phase. This finding highlighted the need for further refinement of the conditions for the analysis of lycopene.

#### 3.1.2. Influence of Operating Parameters

From the evaluation of different stationary and mobile phases, it was evident that the use of BEH 2EP as the stationary phase along with CO2/MeOH:: 85:15 (*v*/*v*) as mobile phase was the most suitable to ensure effective elution and separation of lycopene. The same was therefore considered for optimization of other operating parameters, such as mobile phase flow rates (0.5, 1.0, and 1.5 mL/min), ABPR (1600, 1800, and 2000 psi), and column temperature (35, 45, and 55 °C).

The mobile phase’s flow rate was reported to affect the density inside the column, which consequently affected the retention factors [22,34]. Increased flow rates, therefore, can improve the column performance and lead to reduced tR. As envisaged, tR and T values decreased with the increase in the flow rate. However, k and N values increased with the rise in the flow rate from 0.5 to 1 mL/min (Table 2) indicating the increase in the efficiency of the column for effective extraction and accurate detection of lycopene. The values decreased with further increase in the flow rates from 1.0 to 1.5 mL/min.

The operating pressure and column temperature directly affected the density of mobile phases used in chromatography. However, the effect is reported to reduce when an organic modifier is added to the CO_2_ based mobile stages [22,34]. An increase in pressure from 1600 to 2000 psi decreased the tR values by 16.03%. Further increase in pressure to 2000 psi reduced tR values by an additional 21.56% (Table 2). The peak tailing factor decreased with increased pressure from 1600 to 1800 psi but increased with further increase in pressure beyond 1800 psi. K and N values increased with an increase in pressure up to 1800 psi and decreased after that. A similar trend was also observed when the column temperature was varied between 35 and 55 °C. The column temperature of 55 °C resulted in a broad peak with splitting. T value significantly increased, whereas k and N values reduced with the increase in temperature from 45 to 55 °C. Ethanol percentage of 15.5%, pressure of 1500 psi, and temperature of 40 °C was found to significantly affect the separation of lycopene, β- carotene, coenzyme Q10, and lutein from palm oil. The retention of the bioactive compounds was mostly dependent upon the percentage of ethanol along with pressure, followed by temperature, respectively [33].

Studies on carotenoids mixture from dietary supplements using the combinations of different parameters, i.e., back pressures (11.0, 13.8, 15.2, 17.9, and 19.3 MPa), temperatures (20 °C, 25 °C, 30 °C, 35 °C), and flow rates (0.8 mL/min, 1.0 mL/min, 1.5 mL/min) showed that with the increase in pressure and flow rate, retention time of the carotenoids was shortened with no change in sensitivity of separation [31].

Recent study on enantioseparation of 27 biologically active basic compounds was conducted using ultra-high performance supercritical fluid chromatography, which showed that the mobile phase additives, especially bases (or the mixture of base and acid), improve peak shape and enhance enantioresolution [44].

### 3.2. Method Validation

The proposed method was validated following a conventional validation procedure that included the following attributes: linearity, limits of detection (LOD) and quantification (LOQ), precision and accuracy, selectivity, recovery, and robustness using lycopene standards [45,46,47].

#### 3.2.1. Calibration Parameters, Limits of Detection (LOD), and Quantification (LOQ)

**Figure 2:**
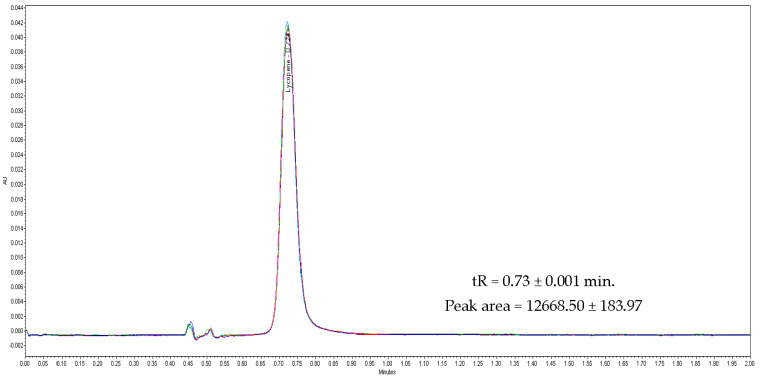
Overlay chromatogram of six injection of lycopene standard (40mg/L) in specificity process.

**Figure 3:**
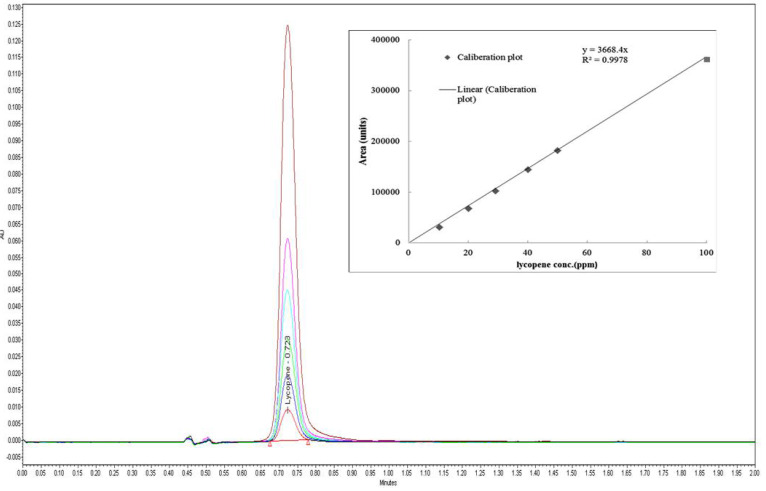
Overlay chromatogram of lycopene standard at different concentration (10, 20, 30, 40, 50, and 100mg/L) in linearity process.

Specificity of the developed method obtained using six injections of a standard stock solution containing 40 µg/mL yielded mean tR values of 0.73 ± 0.001 min. The peak area of 12668.5 ± 183.97 (Figure 2) clearly depicted that the developed method was a good measure for detecting lycopene in the injected sample. A standard curve was assayed over a concentration range of 10 to 100 µg/mL for validation of the linearity method. Data on the correlation between peak areas versus lycopene concentrations were statistically processed by the linear least squares regression analysis method, which resulted in an acceptable correlation coefficient of 0.998 (Figure 3). Moreover, the maximum deviation between the actual concentrations injected, and the concentrations recovered were within ±2.5%. The recovery was performed by spiking a blank supplement sample with lycopene at five levels of concentration with six replications for each concentration (Table 3). Satisfactory recoveries between 97.38% and 102.67% with RSD values lower than 2.5% were achieved.

The limit of detection (LOD) was considered the lowest standard concentration in the analytical run, and it afforded accuracy and precision of at least 20%. In this method, it was 0.14 µg/100 mL. The limit of quantification (LOQ) was calculated as 0.37 µg/100 mL. It showed that the developed method has an effective LOD and LOQ for the lycopene in food matrices.

The mean recoveries were found to be in between 97.3% to 109% in ready-to-feed nutritionals, and the LOQs values of the bioactive compounds were evaluated to be 0.4, 0.1, and 0.3 µg/100 g, respectively [48].

#### 3.2.2. Intra- and Inter-Day Precision and Accuracy

Precision is the parameter that showcases the repeatability of the samples. Here, precision of the developed method was determined by analyzing six different concentrations of lycopene (Table 4). The intra-day precision was determined by injecting these six test solutions six times each on the same day. The inter-day precision determination involved an average of six measurements of the intra-day precision values taken on seven days over three months. Intra-day RSD values were less than 2.5%, whereas the inter-day RSD values were within 4%.

Accuracy is expressed as the percentage recovery of the product. It explains the closeness/nearness of the measured value when compared with the actual value of the product. It was determined at five different concentrations of 50, 80, 100, 120, and 150 ppm of the product and the percentage (%) recovery ranged from 98.96% to 100.99% (Table 3). The results endorsed the potential utility of the proposed method for the analysis of lycopene. Therefore, the procedure developed can be used in quality control, routine investigations, and lycopene stability studies.

Schimpf et al. [48] found that the repeatability of total lycopene ranged from 3.01 to 6.37 and that intermediate precision ranged from 4.29% to 10.3%, in infant and adult nutritional matrixes having the concentrations of >1 μg/100 g.

#### 3.2.3. Robustness

**Figure 4:**
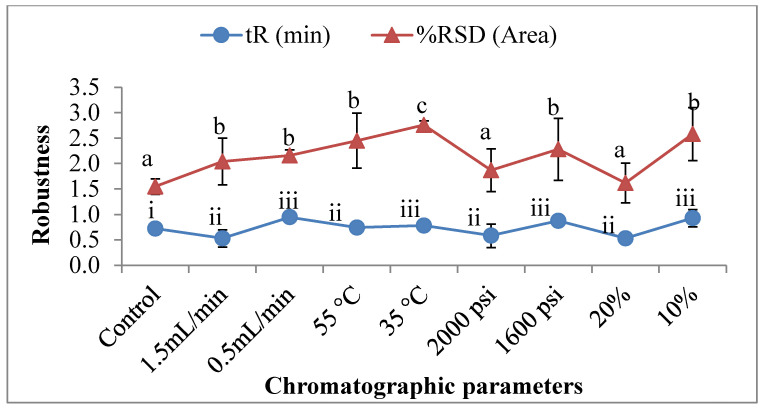
The variation in the system suitability with respect to the robustness parameters (tR and percentage (%) RSD of the area). Control parameters (flow rate—1 mL/min; column temperature—45 °C; ABPR—1800 psi; and modifier concentration—15%). (Values with different letters in the same graph are significantly different with *p* < 0.05).

The method’s robustness was examined by replicating injections (*n* = 6) of 50 µg/mL standard solutions with slight modifications on the chromatographic parameters (flow rate, column temperature, ABPR, and co-solvent). The percentage (%) RSD of peak area after changing the optimized parameter was found to be a significantly influential factor. Except for the changes in ABPR and modifier concentration, the changes in the optimized parameters were substantially taken care of. The most notable factors that were found to influence the percentage (%) RSD of control parameters included the column temperature and modifier concentration, which resulted in a percentage increase of up to 54.65% and 41.92%, respectively, and a shift in the RSD values from 1.55% to 2.75% and 2.58% at 35 °C and 10% of organic solvent (Figure 4). Similarly, tR also increased significantly from 0.725 min to 0.948, 0.874, and 0.931 min at the 0.5 mL/minutes of flow rate, ABPR of 1600 psi, and 10% organic concentration CO_2_, resulting in an up to 30% increase in the total tR values.

### 3.3. Analysis of Lycopene Extracted from Different Matrices

**Figure 5:**
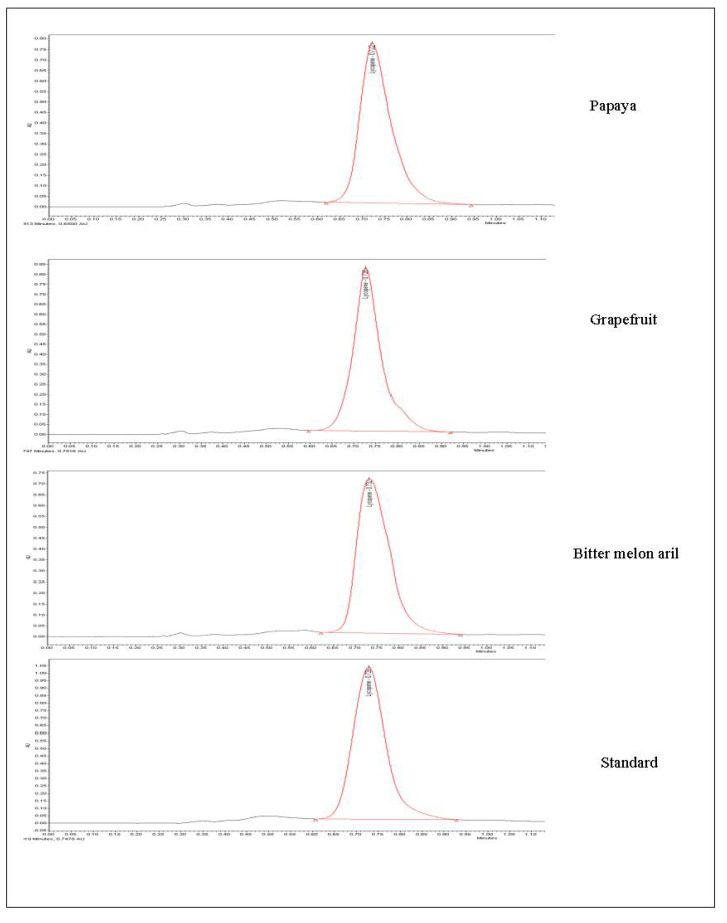
Evaluation of the developed method for determination of lycopene extracted from different matrices.

The developed method was used to quantify lycopene content from papaya, grapefruit, and ripe bitter melon aril. Figure 5 shows the UPSFC spectra of the respective samples, including standard lycopene at 0.72 min. The lycopene content in the fresh papaya, grapefruit, and aril was 14 ± 1.28, 9.8 ± 0.63, and 215 ± 4.83 mg/100 g. The developed UPSFC-DAD method had good recovery of 100.32%, LOD of 0.14 µg/100 mL, and LOQ of 0.37 µg/100 mL (Table 5). Satish et al. [16] have developed an HPLC method for the quantification of lycopene. It reported a retention period of 14.2 min. However, recently, Figueira et al. [49] have developed a quick UHPLC-PDA method for quantifying lycopene that reduced the tR to 2.0 min from a total run period of 4.5 min. The technique illustrated that UHPLC-PDA could be a reliable and effective method for determining lycopene, although using the 100% organic solvents is a significant concern. Developed UPSFC-DAD methods provide lycopene capability within 1 min with 80% less organic solvent consumption since 80% of the mobile phase is CO_2_.

## 4. Conclusions

Supercritical fluid-based based UPSFC-DAD is a rapidly increasing technique in several pharmaceutical or forensic applications. Nonetheless, the analysis of individual carotenoids remains a challenging task. In this study, UPSFC-DAD methods suitable for the separation and detection of lycopene were developed and optimized. The rapid isocratic UPSFC-DAD process using BEH-2EP—2.1 × 150 mm, 5 µm column, developed for determination and quantification of lycopene from agricultural produce, was fast, reliable, selective, and economical with a good recovery of 100.32%, LOD of 0.14 µg/100 mL, and LOQ of 0.37 µg/100 mL. The requirement of significantly fewer solvents reduces the waste generated and makes it green, eco-friendly, and relatively cost-effective, compared with the prevalent HPLC methods. Significantly reduced retention times combined with high specificity, linearity, robustness, and extremely low LOQ and LOD values make the developed technique more suitable for continuous quality control at the commercial level.

## Figures and Tables

**Table 1:** Results of retention times and chromatographic parameters obtained using different column chemistries and mobile phase combinations.

Column	Co-Solvent	CO_2_/Co-Solvent	*tR* (min)	*T*	*k*	*N*	*HEPT*	*h*
BEH 1.7		100:0	0.657	11.254	0.321	2045	48.90	17.57
	Ethanol	90:10	0.578	10.863	0.425	3456	28.93	10.31
		85:15	0.547	10.547	0.357	3245	30.81	10.89
		80:20	0.527	8.354	0.2401	3578	27.95	9.86
	Methanol	90:10	0.491	5.719	0.445	3012	33.20	11.74
		85:15	0.459	3.725	0.554	3756	26.62	9.43
		80:20	0.320	4.154	0.521	3456	28.96	10.22
HSS C_18_		100:0	0.887	6.781	0.157	1837	54.44	19.37
	Ethanol	90:10	0.794	6.254	0.424	2245	44.54	15.74
		85:15	0.775	5.724	0.456	2578	38.79	14.57
		80:20	0.548	7.587	0.357	2012	49.70	17.68
	Methanol	90:10	0.654	5.457	0.162	3156	31.69	12.51
		85:15	0.524	5.024	0.181	3356	29.80	10.76
		80:20	0.457	4.578	0.215	3358	29.78	11.27
BEH 2EP		100:0	1.029	5.387	0.387	4198	35.73	12.63
	Ethanol	90:10	1.014	4.124	0.524	4548	32.98	11.67
		85:15	0.937	3.475	0.451	4864	32.72	11.56
		80:20	0.787	5.015	0.658	5257	28.53	10.14
	Methanol	90:10	0.974	3.875	0.571	8948	16.76	5.92
		85:15	0.722	2.271	0.672	13367	11.22	3.97
		80:20	0.524	2.712	0.587	11078	13.54	4.78

Note: Run time—2 min; ABPR—1800 psi; sample temperature—20 °C; column temperature—45 °C; detector wavelength—434 nm; and mobile phase flow rate—1 mL/min. tR– retention time; T—tailing factor; k—retention factor; N—number of theoretical plates; HEPT—height equivalent to theoretical plate; and h—reduced plate height.

**Table 2:** Results of retention times and chromatographic parameters obtained under varying operating conditions using BEH 2EP column and CO_2_/MeOH: 85:15 as the mobile phase.

**Flow Rate (ml/min)**	***tR*** **(min)**	***T***	***k***	***N***	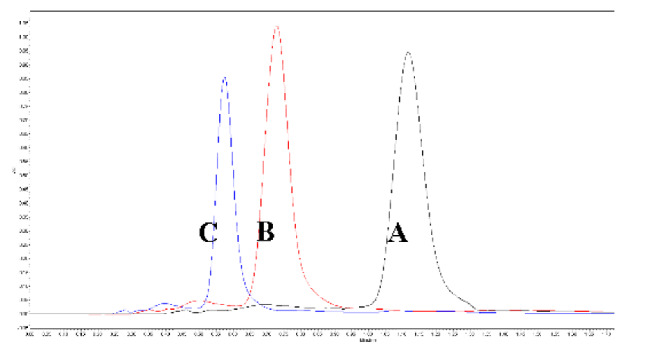
0.5 (A)	0.957	3.473	0.527	12618	
1.0 (B)	0.723	2.249	0.668	13418	
1.5 (C)	0.520	2.189	0.685	12015	
Run time—2 min.; ABPR—1800 psi; sample temperature—20 °C; column temperature—45 °C; and detector wavelength—434 nm					
ABPR (psi)	*tR* (min)	*T*	*k*	*N*	* 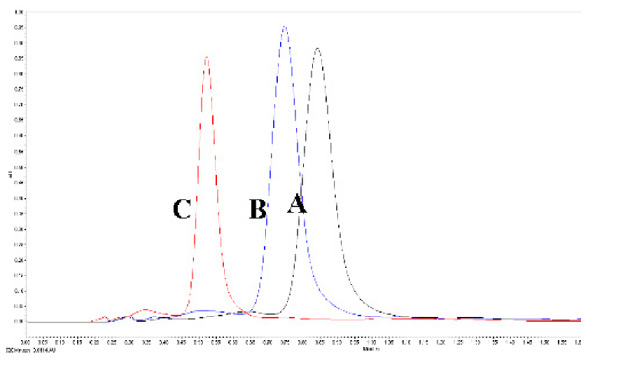 *
1600 (A)	0.867	3.409	0.366	12701	
1800 (B)	0.728	2.152	0.651	13618	
2000 (C)	0.571	2.453	0.708	12603	
Run time—2 min.; sample temperature—20 °C; column temperature—45 °C; detector wavelength—434 nm; and mobile phase flow rate—1 mL/min.					
Column temp (°C)	*tR* (min)	*T*	*k*	*N*	* 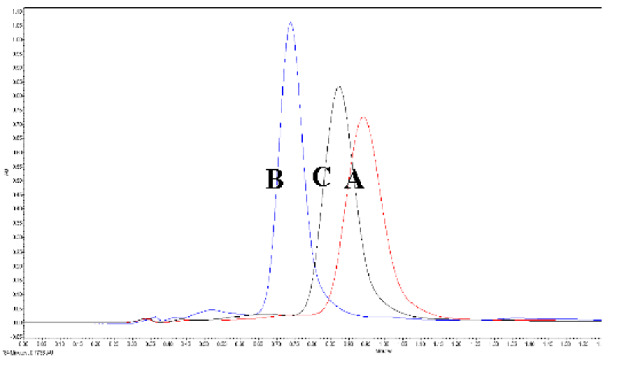 *
35 (A)	0.767	2.911	0.745	11968	
45 (B)	0.724	2.231	0.622	13574	
55 (C)	0.738	4.895	0.525	12921	
Run time—2 min; sample temperature—20 °C; column temperature—45 °C; detector wavelength—434 nm; and mobile phase flow rate—1 mL/min.					

*tR*—retention time; *T*—tailing factor; *k*—retention factor; *N*—number of theoretical plates.

**Table 3:** Recovery (%) of lycopene at different dilutions of 40 ppm stock solution (*n* = 6).

Concentration (%)	Recovery (%)	Mean	SD	%RSD
50	100.32	99.58	0.52	0.64
80	102.67	100.99	2.36	1.98
100	101.45	100.91	0.77	0.64
120	97.38	98.96	2.22	2.44
150	98.56	99.07	0.72	0.93

**Table 4:** Data for in system precision process of lycopene validation (*n* = 6).

Concentration (µg/mL)	Intra-Day Recovery (%)			Inter-Day Recovery (%)		
	Mean	SD	%RSD	Mean	SD	%RSD
10	100.02	1.27	1.57	99.23	1.27	2.87
20	99.18	0.27	1.85	98.75	0.61	1.57
30	98.78	0.84	2.12	100.00	2.08	3.87
40	99.27	0.85	1.27	98.82	0.87	2.82
50	100.7	3.18	2.28	99.51	0.55	4.18
100	99.98	1.22	3.28	100.28	1.32	2.84

**Table 5:** A comparative table of total run time, percentage (%) recovery, LOD, and LOQ for the lycopene using UPSFC-PDA and other LC methods.

Method	Run Time (minutes)	Compounds	Recovery (%)	LOD (µg/100 mL)	LOQ (µg/100 mL)	References
UPSFC-PDA	2	Lycopene	100.32	0.14	0.37	current method
HPLC	20	Lycopene	-	0.50	1.00	[16]
UHPLC		Lycopene	92.8	0.24	0.80	[49]
HPLC	30	Lycopene	81.70	1.56	3.90	[50]
LC-DAD	30	Lycopene	102.59	0.05	NA	[51]
HPLC	10	Lycopene	77.00	0.10	0.30	[52]
UHPLC	30	Lycopene	110.00	0.09	0.29	[53]
HPLC	14	Lycopene	>97.00	0.01	0.04	[54]

## Data Availability

Data supporting reported results are available upon request.
